# Impact of the COVID-19 pandemic on seasonal influenza in the WHO Western Pacific Region, 2016–2024

**DOI:** 10.5365/wpsar.2025.16.1230

**Published:** 2025-07-04

**Authors:** Jozica Skufca, Victoria Katawera, Phuong Nam Nguyen, Tamano Matsui, Babatunde Olowokure, the Western Pacific Regional Office Health Emergencies Programme Team

**Affiliations:** a World Health Organization Regional Office for the Western Pacific, Manila, Philippines.

Epidemics and pandemics of infectious diseases caused by human respiratory viruses, mainly influenza viruses and coronaviruses, represent a significant global threat to public health, societies and economies. The most recent threat was first detected in 2019 and was caused by a novel coronavirus, severe acute respiratory syndrome coronavirus 2 (SARS-CoV-2), which spread rapidly, leading to its characterization as a pandemic. The World Health Organization (WHO) declared COVID-19, the disease caused by SARS-CoV-2, a public health emergency of international concern on 30 January 2020 and a global pandemic on 11 March 2020. (*1*) Unprecedented and wide-ranging public health measures were implemented globally in response to COVID-19. This perspective paper describes seasonal influenza activity in the WHO Western Pacific Region from 2016 to 2024, assesses the impact of the COVID-19 pandemic on influenza trends and considers implications for future surveillance strategies.

The COVID-19 pandemic interrupted the typical timing, intensity and duration of seasonal influenza activity, resulting in significant declines in seasonal influenza virus detection globally and in the Region. (*2*-*4*) This descriptive analysis draws on data from laboratory-confirmed cases of influenza reported to the WHO Global Influenza Surveillance and Response System (GISRS) via its FluNet platform, (*5*, *6*) as well as information from seasonal influenza situation reports produced by the WHO Regional Office for the Western Pacific on a biweekly basis. (*7*)

As of December 2024, the WHO Western Pacific Region comprised 37 countries and areas (hereafter referred to as countries), including 21 Pacific island countries and areas (PICs). The Region is highly diverse: it includes one of the world’s most populous countries and some of its smallest; both low- and high-income countries; and several of the world’s largest megacities as well as some of the most remote island communities. Countries also vary widely in climate, social and cultural characteristics, and—most notably for the purposes of this paper—in their influenza surveillance capabilities. Some countries have established advanced sentinel syndromic surveillance influenza systems that generate epidemiological and virological data critical for identifying and continuously monitoring antigenic changes in influenza viruses. These systems use the syndromic case definitions for influenza-like illness (ILI), severe acute respiratory illness (SARI) and, in some countries, acute respiratory infection (ARI). Other countries in the Region have more modest surveillance systems and/or rely more heavily on routine indicator-based surveillance methods. Across the Region, 21 national influenza centres in 15 countries are currently part of the GISRS laboratory network and are actively engaged in establishing laboratory surveillance for influenza and other respiratory viruses in their respective countries. As of December 2024, a total of 17 Western Pacific countries reported virological surveillance data to FluNet (including two PICs), and 26 countries reported epidemiological data to FluID (including 20 PICs).

Available FluNet data for the Region are shown in **Fig. 1**, demonstrating that during the height of the COVID-19 pandemic (2020–2021), the incidence of laboratory-confirmed influenza was less than in previous years (2016–2019). In the pre-pandemic period (from October 2016 to October 2019), the reported influenza positivity rate varied seasonally between 1.9% (229/12 181) and 40.0% (9070/22 675), but decreased sharply to below 1% (range: 0–214 positive samples/0–21 400 tested samples) from March 2020 to April 2021 (**Fig. 1**). Influenza cases reported in Japan in the 2020/2021 winter, for example, were estimated to be less than 1/1000 of the typical annual number of cases. (*8*)

**Fig. 1:**
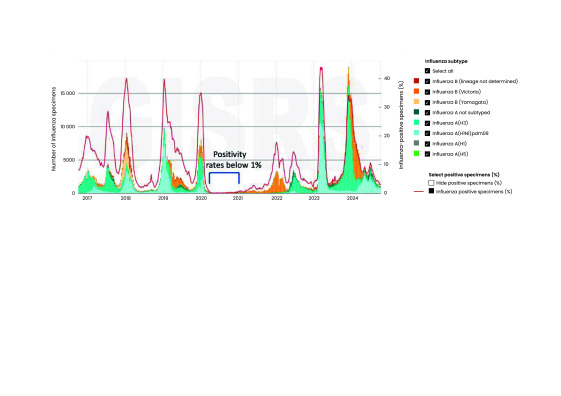
Number of specimens positive for influenza by subtype, WHO Western Pacific Region, from 7 October 2016 to 7 October 2024

[**insert** Figure 1]

The sharp decline in influenza virus circulation during the COVID-19 pandemic was particularly noticeable in countries with temperate climates. Australia, Japan, New Zealand and Republic of Korea reported the largest declines. (*7*) In other countries, such as Malaysia and Viet Nam, a smaller decline was observed. (*7*) Among the PICs, a decline was observed in Fiji, New Caledonia and Papua New Guinea. (*7*) In the tropical and subtropical PICs, changes were less pronounced but difficult to interpret given that, in these countries, the circulation of the influenza virus is generally low year-round, does not normally exhibit seasonal patterns and peaks are mostly travel-related. (*2*) During 2022, influenza virus activity in the Region was lower than before the COVID-19 pandemic but increased in many Western Pacific countries at the end of 2023 and into the start of 2024 (**Fig. 1**).

Observed reductions in reported influenza cases during the COVID-19 pandemic years have been attributed to multiple factors, (*2*) including the implementation of universal preventive measures (hand sanitization, mask-wearing, physical distancing), as well as reductions in international travel, workplace presence, school attendance and mass gatherings. (*2*-*4*, *9*, *10*) Other possible contributory factors include the promotion of and increase in influenza vaccination (which has been reported in some countries in the Region) and direct viral interference with SARS-CoV-2. (*2*, *3*) It has also been suggested that changes in the epidemiology of influenza viruses may have played a role. Although laboratories continue to test for B/Yamagata lineage viruses, there have been no confirmed detections of B/Yamagata/16/88 lineage viruses since March 2020, suggesting that naturally occurring B/Yamagata viruses are probably no longer circulating in the population. This apparent disappearance may contribute to reduced overall influenza activity, as it removes one of the two major influenza B lineages that previously co-circulated, thereby decreasing the diversity and volume of circulating influenza viruses. Previously, B/Yamagata and B/Victoria lineages co-circulated in variable proportions until March 2020. (*5*)

It seems unlikely that the Region-wide reduction in the incidence of laboratory-confirmed influenza during the pandemic was associated with a lack of testing. Global data in FluNet indicate that, overall, the level of testing was no lower than it had been in pre-COVID-19 years. (*2*) Some countries, such as Australia, even increased influenza testing during the COVID-19 pandemic; however, the positivity rate was much lower than that recorded pre-pandemic. (*11*) Conversely, a meta-analysis concluded that there was a global decline in influenza surveillance during COVID-19; the pooled proportion of samples tested for influenza during the pandemic was 0.48% (95% confidence interval: 0.28–0.68%), compared with 0.69% in 2019 (95% confidence interval: 0.45–0.92%). (*12*) Nevertheless, there were some notable exceptions (for example, Canada). It is plausible that these global analyses are masking national variations, with some countries increasing testing and others scaling back during the pandemic. Some of the reasons for the latter include a shift in priorities and funding away from influenza in favour of COVID-19, the closure of outpatient clinics and the preferential referral of persons with ILI for SARS-CoV-2 testing. In some settings, suspected cases may have been referred to hospitals and, thus, were not captured in clinics as ILI/ARI cases. (*12*) Such factors may well have played out in some of the lower-income PICs in the Region.

The COVID-19 pandemic has emphasized the importance of sustainable influenza surveillance systems for continuous monitoring and reporting of influenza viruses across the Region. Several countries, including some PICs, successfully scaled up their laboratory surveillance capacity during the pandemic, and many have already integrated existing surveillance systems for testing both influenza and SARS-CoV-2 or are in the process of doing so. While the 2018 Asia Pacific Strategy for Emerging Diseases and Public Health Emergencies (APSED) has been a valuable resource, (*13*) the COVID-19 pandemic has identified areas that need further work. The new Asia Pacific Health Security Action Framework builds on the foundations of APSED, urging countries to establish resilient health systems to prevent, rapidly detect, be ready for and effectively respond to outbreaks with pandemic potential. It also advocates for multisource surveillance through the integration of multiple sources to strengthen early detection and response to public health threats. (*14*)

The detection of influenza through traditional sentinel surveillance for ARI/ILI/SARI may change due to health-care-seeking behaviours and other compounding factors in emergencies such as those seen during the COVID-19 pandemic. These limitations highlighted weaknesses in relying solely on sentinel systems and underscored the need for complementary data sources. No single-source surveillance system can provide a complete picture of the situation. A multisource surveillance system approach is, therefore, essential for providing comprehensive insights on influenza activity and severity by integrating laboratory, event-based surveillance systems, indicator-based surveillance, as well as multiple indicators such as test positivity, hospitalizations, intensive care unit (ICU) admissions and mortality estimates to monitor severity. In addition to strengthening national systems, timely regional and international information sharing is critical in detecting, assessing risk and responding to emerging threats.

The WHO Regional Office for the Western Pacific is working closely with countries in the Region to strengthen existing sentinel surveillance systems; build sustainable laboratory systems; streamline and enhance timely data reporting; integrate laboratory and epidemiological systems on COVID-19, influenza and other respiratory pathogens; conduct surveillance for zoonotic events; and promote modelling and use of new data sources to improve understanding of severity. By analysing trends in indicators such as hospitalizations, ICU admissions and deaths, it is possible to better forecast epidemics and pandemics, even when case reporting is limited. Such activities contribute to the continuous monitoring of influenza virus evolution and the building of resilient systems at national and subnational levels. This will facilitate improved preparedness, readiness, response and resilience to potential future pandemics caused by respiratory viruses.

## References

[1] Gupta S, Gupta T, Gupta N. Global respiratory virus surveillance: strengths, gaps, and way forward. Int J Infect Dis. 2022 Aug;121:184–9. 10.1016/j.ijid.2022.05.032 35584744 PMC9107382

[2] Karlsson EA, Mook PAN, Vandemaele K, Fitzner J, Hammond A, Cozza V, et al. Review of global influenza circulation, late 2019 to 2020, and the impact of the COVID-19 pandemic on influenza circulation. Wkly Epidemiol Rec. 2021;25:241–64. [cited 2025 Jan 10] Available from https://www.who.int/publications/i/item/who-wer-9625-24-264

[3] Chow EJ, Uyeki TM, Chu HY. The effects of the COVID-19 pandemic on community respiratory virus activity. Nat Rev Microbiol. 2023 Mar;21(3):195–210. 10.1038/s41579-022-00807-9 36253478 PMC9574826

[4] Bonacina F, Boëlle PY, Colizza V, Lopez O, Thomas M, Poletto C. Global patterns and drivers of influenza decline during the COVID-19 pandemic. Int J Infect Dis. 2023 Mar;128:132–9. 10.1016/j.ijid.2022.12.042 36608787 PMC9809002

[5] Global Influenza Surveillance and Response System (GISRS) [Internet]. Geneva: World Health Organization; 2025. Available from: https://www.who.int/initiatives/global-influenza-surveillance-and-response-system, accessed 18 February 2025.

[6] Global Influenza Programme. FluNet [Internet]. Geneva: World Health Organization; 2025. Available from: https://www.who.int/toolkits/flunet, accessed 10 January 2025.

[7] Seasonal influenza situation reports. Manila: WHO Regional Office for the Western Pacific; 2024. Available from: https://www.who.int/westernpacific/wpro-emergencies/surveillance/seasonal-influenza, accessed 10 January 2025.

[8] Takahashi H, Nagamatsu H, Yamada Y, Toba N, Toyama-Kousaka M, Ota S, et al. Surveillance of seasonal influenza viruses during the COVID-19 pandemic in Tokyo, Japan, 2018-2023, a single-center study. Influenza Other Respir Viruses. 2024 Jan 5;18(1):e13248. 10.1111/irv.13248 38188373 PMC10767599

[9] Takeuchi H, Kawashima R. Disappearance and re-emergence of influenza during the COVID-19 pandemic: association with infection control measures. Viruses. 2023 Jan 13;15(1):223. 10.3390/v15010223 36680263 PMC9862942

[10] Soo RJJ, Chiew CJ, Ma S, Pung R, Lee V. Decreased influenza incidence under COVID-19 control measures, Singapore. Emerg Infect Dis. 2020 Aug;26(8):1933–5. 10.3201/eid2608.201229 32339092 PMC7392467

[11] Olsen SJ, Azziz-Baumgartner E, Budd AP, Brammer L, Sullivan S, Pineda RF, et al. Decreased influenza activity during the COVID-19 pandemic – United States, Australia, Chile, and South Africa, 2020. MMWR Morb Mortal Wkly Rep. 2020 Sep 18;69(37):1305–9. 10.15585/mmwr.mm6937a6 32941415 PMC7498167

[12] Sabeena S, Ravishankar N, Robin S. The impact of COVID-19 pandemic on influenza surveillance: A systematic review and meta-analysis. Indian J Public Health. 2022 Oct-Dec;66(4):458–65. 10.4103/ijph.ijph_926_22 37039174

[13] Dueger E, Peters L, Ailan L. Marking the 1918 influenza pandemic centennial: addressing regional influenza threats through the Asia Pacific Strategy for Emerging Diseases and Public Health Emergencies. Western Pac Surveill Response J. 2019 Nov 19;9(5) Suppl 1:1–4. 10.5365/wpsar.2018.9.5.000 31832245 PMC6902656

[14] Asia Pacific health security action framework. Manila: WHO Regional Office for the Western Pacific; 2024. Available from: https://iris.who.int/handle/10665/377083, accessed 10 January 2025.

